# Impacts of climate change-induced natural hazards on women and their human rights implications: A study in the southwest coast of Bangladesh

**DOI:** 10.1016/j.jmh.2024.100221

**Published:** 2024-03-06

**Authors:** Md Shamsuddoha, Md. Akib Jabed, Md Shahnul Islam, Naznin Sultana, Al Imran, Sheikh Nur Ataya Rabbi, Tanje Un Jenat, Shanjia Shams, Mir Mehoraf Sharif

**Affiliations:** Center for Participatory Research and Development (CPRD), Dhaka, Bangladesh

**Keywords:** Climate change, Women, Rights violations, Coastal belt, Bangladesh

## Abstract

Women in coastal areas are particularly vulnerable to climate change impacts due to existing gender inequality and socio-cultural norms in Bangladesh. This research aims to explore the vulnerability of women to climate change-induced natural hazards, the challenges they face due to the chain impacts of climate change, and the resulting violation of women's rights. Quantitative and qualitative data were used in this study, where 260 structured questionnaire surveys and 15 Focus Group Discussions were performed at Mongla and Shyamnagar Upazilas in Bagerhat and Satkhira districts, respectively. The quantitative data were analyzed using SPSS 26.0 software, and qualitative data was analyzed thematically. The results disclose that most respondents in Mongla and Shyamnagar reported an increase in the occurrence rate of climate change-induced natural hazards, with cyclones, salinity, and riverbank erosion being the most devastating in Mongla and cyclones, salinity, and storm surges in Shyamnagar. It affects the lives and livelihoods of people, with women being particularly vulnerable due to limited access to education, healthcare, and economic opportunities, and women's rights are violated in these areas. Half of the study area's population has been suffering from infections or inflammation in reproductive organs, especially among fisherwomen. The findings of this study recommend that provisions for alternative livelihoods should be made for women, and all women must be brought under the umbrella of fair social safety net programs.

## Introduction

Bangladesh is one of the few nations on the Earth that best exemplifies the severe unfairness of the climate problem. Although Bangladesh has been a minor contributor to global climate change, with an estimated 0.4 % of the world's total greenhouse gas emissions in 2018, it has shouldered massive death, destruction of resources, and economic loss due to climate change impacts (World Bank (WB) 2022). The occurrence of global climate change can be felt in the form of detrimental climate stresses on the country, for example- shift in rainfall duration, intensity, and pattern; change in seasonal variation; and the unprecedented increase in extreme sudden and slow onset natural hazards such as cyclones, floods, storm surges, riverbank erosion, droughts, sea level rise, salinity intrusion, and land degradation (Asaduzzaman et al., 2010; Yu et al., 2010; Hossain and Deb, 2011). Climate change has already brought massive havoc on the lives and livelihoods in Bangladesh's coastal area and many arid and semi-arid regions (Ministry of Planning (MoP) 2011).

Although everyone is affected by climate change, not every person faces the impacts equally. An individual's vulnerability to climate change impacts can be shaped by, among other things- the individual's location of residence, race, gender, age, culture, ethnicity, and other socioeconomic determinants. Among these, the gender perspective of climate change impacts is of significant concern because, in a particular socioeconomic, religious, and cultural setting, gender relations serve as the foundation for the roles and status of men and women (Hai and Smyth, 2012). This implies that the degrees and types of vulnerabilities women and men face to climate change-induced natural hazards, particularly those brought on by climate change, differ (Hai and Smyth, 2012). In Bangladesh, many women are more vulnerable than their male counterparts to climate change-induced natural hazards due to their gender identity (Juran and Trivedi, 2015). Here, women are more in danger from natural catastrophes than males are right away, and they are less resilient and have less agency in the wake of climate change-induced natural hazards as a direct consequence of the weight of gender roles, inequalities, including power limitations that they already bear (Juran and Trivedi, 2015).

During significant climate change-induced natural hazard events, women's mortality rates often superseded men's by multiple times. For instance, in the cyclone and floods in 1991, the death rate for women was as good as five times higher than men's (Sharmin and Islam, 2013). Likewise, cyclone Sidr in 2007 and tropical storm Mahasen in 2013 claimed women's lives more than men's (Schmuck, 2002; Bhuiyan, 2013; Alam and Rahman, 2014). The picture behind this scene is that many women prefer to stay behind at home rather than go to emergency shelters due to restrictive societal norms, lack of lifesaving skills like swimming and climbing, attempting to save children and possessions, lousy transportation and communication, pregnancy or disability, previous experience of harassment or other issues at shelter, etc. (Sultana, 2014; Hossain et al., 1992).

In addition to the other effects of climate change-induced natural hazards, women face increased rates of sexual and domestic violence during and after climate change-induced natural hazards (Rahman, 2013). For instance, due to increased salinity in the southwest coastal belt of Bangladesh, women and adolescent girls who have been compelled to fetch potable water from remote places often encounter sexual harassment. Their physical and mental stress also increases, but being a caregiver, they cannot avoid it (Imran et al., 2022). Given their heightened susceptibility to climate change-induced natural hazards, households seem to have turned to child marriage as a coping mechanism. According to a recent study conducted in four coastal districts of Bangladesh, there is a positive association between shocks related to natural disaster events and the incidence of child marriage and other factors like economic vulnerability, patriarchal norms, etc. (Asadullah et al., 2021).

Increased intensity and frequency of devastating climate change-induced natural hazards have disproportionate impacts not only on the physical but also on the mental health of women (Goodman, 2016). Insufficient facilities for women, especially for pregnant and breastfeeding mothers, unhealthy sanitation, lack of safe drinking water, and inadequate medical facilities make women suffer vile consequences during their shelter stay (Nur et al., 2021). Given that women often lack adequate nourishment, it makes sense that water-borne illnesses would affect them more frequently (Rahman, 2013). Additionally, drinking salt water during pregnancy increases the risk among women of pre-eclampsia, hypertension, and newborn mortality owing to a shortage of freshwater supplies (Shammi et al., 2019). Due to their distinct dietary requirements (especially during pregnancy or nursing) and the fact that they are often ranked lower in the home food hierarchy than males, women are more likely to experience malnutrition (Rahman, 2013).

The full and effective enjoyment of the human rights entrenched in the Universal Declaration of Human Rights (UDHR) and other international human rights instruments is severely affected by climate change. Women constitute around half of Bangladesh's total population and are inherently entitled to all human rights on an equal basis as men, according to the Constitution of Bangladesh (Government of the People's Republic of Bangladesh, 1972). However, all the impacts of climate change-induced natural hazards on women discussed above are straightforward depictions of violating those rights. All people have the right to a social and international environment where their rights and freedoms can be fulfilled, as stated in Article 28 of the Universal Declaration of Human Rights (UNHRC, 1948). The adverse effects of climate change put this order and everyone's rights and liberties in danger. According to a resolution of the Human Rights Council (A/HRC/RES/41/21), climate change affects several cultural rights, rights to life, self-determination, development, health, food, water, sanitation, and sufficient housing. Along with the International Covenant on Economic, Social, and Cultural Rights (ICESCR), the International Covenant on Civil and Political Rights (ICCPR) and the Convention on the Rights of the Child (CRC), the 1979-adopted Convention on the Elimination of All Forms of Discrimination Against Women (CEDAW) provides a valuable framework for relating climate change to women's protection from harms brought about by climate-related vulnerabilities as well as to the advancement of gender equality, including women's capacity to lead alongside men in the pursuit of sustainable solutions (United Nations, International Covenant on Economic, Social, and Cultural Rights, OHCHR, 1966; United Nations, 1966; United Nations 1989; United Nations, 1979; Alam et al., 2015).

This research intended to explore the diverse and disproportionate climate change vulnerabilities and impacts on women and their implications for human rights violations throughout the impacts chain extended to secondary and tertiary levels. Earlier research has primarily focused on women's health issues; however, no such extensive research has been found about violating women's rights due to the chain impacts of climate change in Bangladesh. Considering women's vulnerability and the profoundness of their sufferings and loss and damage situation, this research was conducted at three Unions of Mongla Upazila (i.e., Chila Union, Chandpai Union, and Sunderban Union) in Bagerhat district and three Unions of Shyamnagar Upazila (i.e., Munshiganj Union, Burigoalini Union and Gabura Union) in Satkhira district.

The specific objectives are:

a) To explore the vulnerability of women due to the increased frequency and intensity of natural hazards,
b) To understand the challenges women face due to the continued impacts of climate change-induced natural hazards,
c) To comprehend the issue- violation of women's rights due to climate change-induced natural hazards.

## Materials and methods

Bangladesh is widely vulnerable to various natural hazards, such as geographically disadvantaged locations, colossal populations, poverty, weak governance, etc. Climate change is acting like a cherry on the top, making natural hazards more frequent and intense. Although Bangladesh seems to be a small country with only 144,570 km^2^, its physical geography is diverse, and different parts are prone to distinct calamities. This fact is more pronounced for the climate change impacts, as the most tangible effects of climate change felt in Bangladesh are frequent and intense tropical cyclones and associated storm surges, which are formed in the Bay of Bengal and make landfall in the coastal zone of Bangladesh. Before conducting this study, a rigorous secondary literature review was done to find out which part of the country is facing more drastic impacts of climate change, and the results guided us towards the southwest coastal region of the country. Besides, three pilot studies were conducted in three different parts of the country, namely- the north-eastern part (Sunamganj district), the north-western part (Rajshahi district), and the southwestern part (Bagerhat and Satkhira district). Among all the areas, the southwestern part was found to be suffering from the most pronounced climate change impacts, and women in this area are bearing the brunt more severely than other parts of the country.

After narrowing down the study area, we tried to understand the impacts of climate change-induced natural hazards on coastal women and their implication for human rights violations. An individual's vulnerability to climate change impacts is also shaped by the intersectionality of that individual's different identities, such as gender, economic condition, occupation, residence, and so on. Thus, men and women in the study area are impacted differently. Again, not all individuals of a gender are affected the same. Considering this, we chose our study population to be women (including adolescent girls aged 14–18) from coastal areas more vulnerable to climate change-induced natural hazards. Some of the inclusion criteria were more susceptible individuals, individuals associated with fishing as an occupation, individuals living at a risky location, individuals who have experienced several devastating natural hazards, individuals who have been displaced due to natural hazards, etc. We avoided taking responses from well-off communities, individuals with secured households and stable livelihood options, etc. As the field study focuses on collecting primary data on the vulnerability of women to climate change impacts, we defined vulnerability as the propensity or predisposition to be adversely affected, which shows peoples’ sensitivity or susceptibility to harm and lack of capacity to cope and adapt. We crafted our questions to capture individuals’ vulnerability, sensitivity to natural hazards, and ability to manage and adjust to post-disaster situations. For example, an individual's educational and economic condition, occupation, etc., shows their susceptibility toward frequent and intense natural hazards. Being pregnant, wearing clothing that prevents quick movement, being unable to influence household-level decisions, etc., shows women's sensitivity to being adversely affected by hazards. Loss and damage from recurrent disasters, not having solid and reliable social security, water insecurity, and illnesses associated with occupation undermine women's coping and adaptation capacity against natural hazards. Qualitative and quantitative data were collected, and data triangulation was done to generate results.

### Study area

**Fig. 1:**
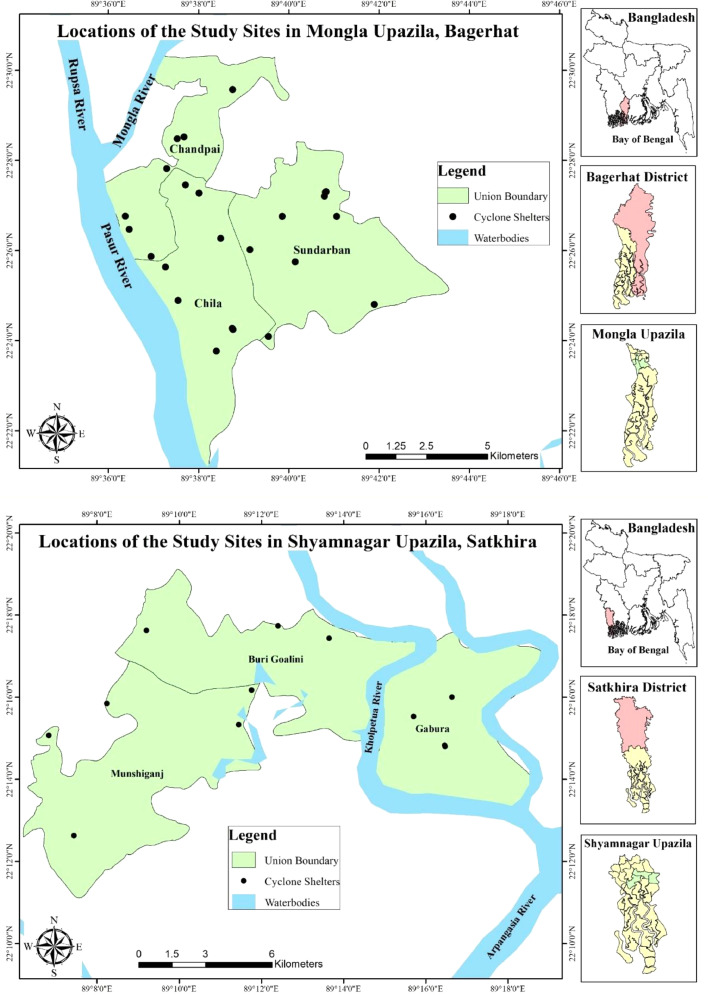
Location of the Study areas.

Based on desk research and pilot study, we selected two Upazilas from two districts of the southwest coast of Bangladesh, both of which are the southernmost parts of the country. From the pilot study, we found a significant difference in women's responses regarding their problems caused by the hazards. However, similar climate change-induced hazards were prevalent in both studied upazilas. To unveil the different locational impacts on women from natural hazards, we collected data from two different upazilas and chose six unions (A Union is the smallest rural administrative and local government unit in Bangladesh). Considering the budget, human resources, time, and other factors, we collected the primary data for this study from three unions of each Upazila, namely Chila, Chandpai, and Sundarban unions of Mongla Upazila and Burigoalini, Munsiganj, and Gabura unions of Shyamnagar Upazilas (Fig. 1). All of these unions are situated at the edge of the Sundarban mangrove forest which is a UNESCO world heritage site. The area is characterized by its low-lying plain and tidal river systems, which lie about 1–3 meters above the mean sea level (Mondal et al., 2013). The Sundarbans provide a vital ecosystem service by protecting the region from devastating tidal surges and cyclones and offering livelihoods to millions living there. The areas of Mongla and Shyamnagar Upazila are consecutively 361,072 and 486,360 acres. According to the Bangladesh Bureau of Statistics (Bangladesh Bureau of Statistics (BBS), 2011), the population of Mongla Upazila is 136,588 (Males 71,492 and female 65,096) with a literacy rate of 57.20 %, and the population of Shyamnagar Upazila is around 318,254 (Male 153,441 and Female 164,813), with a literacy rate of 48.6 %. Both areas are predominantly inhabited by people who rely on agriculture, fishing, fisheries, and other occupations based on the Sundarbans mangrove forest for their livelihoods.

### Data collection

To fulfill research objectives, we planned to follow a mixed-method approach. In doing so, we collected both quantitative and qualitative data. After analyzing quantitative and qualitative data separately, methodological triangulation was conducted.

For quantitative data collection, we used a structured questionnaire to conduct a survey. It was pre-tested through a pilot study. It included close-ended multiple choice/ multiple response questions. The probability sampling method (simple random sampling) was used to collect data. In the case of our study locations, we could not determine the exact number of vulnerable women (i.e., population size is unknown). Thus, the sample size for the questionnaire survey was calculated using the following formula:

(i) $$\text{Sample}\;\text{size}=\frac{z^{2}\times p\times \left(1-p\right)}{MoE^{2}}$$

Where, *z* = *z* score, *p* = Standard Deviation, and MoE = Margin of Error/ Confidence Interval

We calculated the sample size with a 95 % confidence level (z score = 1.96), 0.5 standard deviation (p), and 10 % margin of error/ confidence interval (MoE).

(ii) $$\begin{array}{c} \text{Sample}\;\text{Size}\;\left(\text{Mongla}\right)=\frac{1.96^{2}\times 0.5\times \left(1-0.5\right)}{{\left(10\%\right)}^{2}}\\ =96\left(Approximately\right) \end{array}$$

(iii) $$\begin{array}{c} \text{Sample}\;\text{Size}\;\left(\text{Shyamnagar}\right)=\frac{1.96^{2}\times 0.5\times \left(1-0.5\right)}{{\left(10\%\right)}^{2}}\\ =96\left(Approximately\right) \end{array}$$

As the calculations derive the minimum number of samples to draw data from to be 96, we planned to collect as much as possible above this number. Finally, we gathered primary data through a questionnaire survey from 143 respondents in Mongla and 117 respondents in Shyamnagar (260 in total). We administered the questionnaire as a household survey (collecting only one response from one household). The questionnaire was pre-tested and validated through a pilot study.

We conducted Focus Group Discussions (FGDs) for the qualitative part of the study. There were 5 FGDs in Mongla and 10 FGDs in Shyamnagar. At the FGDs, open-ended questions were posed to ignite a casual conversation and examine participants’ responses. Questions were of the same theme as the questionnaire survey, so the responses could complement each other. The participants of the FGDs were also selected purposively to represent the marginalized, impoverished, and most sufferers of the impacts of natural hazards. There were 10–15 participants in each FGD, and the discussions took place for around 1 hour. The FGD sessions were recorded and transcribed for further analysis.

Many existing scientific literature and national and international policies/ laws were reviewed to create a solid basis for analyzing women's rights violations in the study region.

### Data analysis

For quantitative data analysis, the responses in the questionnaire survey were entered into SPSS 26.0 software for analysis. The descriptive statistics were calculated for frequencies and percentages. The chi-square test was conducted to find the association between some selected attributes of respondents and their responses.

For the qualitative part of the study, the FGD transcripts were analyzed thematically. The similar thematic portions of each FGD were put together to produce a generalizable output.

## Results

### Socioeconomic profile of the respondents

All respondents of this study were female, representing individual families. In this study, respondents were categorized into four different age groups from 14 to above 60 years, where most of the responses came from the 35 to 60 years age group in both areas (51 % in Mongla and 56.4 % in Shyamnagar) (Table 1). In Mongla, the highest percentage (40.6 %) of respondents reported having completed primary education, while in Shyamnagar, the highest portion (47 %) of respondents did not receive any formal education in their lifetime. The average monthly income of most of the households in Mongla (36.4 %) is BDT (Bangladeshi taka (1 USD = 109.31 BDT)) 5000–10,000, and in Shyamnagar (35 %) is BDT 10,000–20,000. Apart from homemaking, the most prominent occupation pursued by the respondents is fishing at nearby rivers (58.7 % in Mongla and 41.9 % in Shyamnagar). Fisherwomen in both study areas have been found to catch mostly shrimp fingerlings wading into waist-deep saline river water with hand-drawn nets.

**Table 1:** Socioeconomic profile of the respondents.

Variable	Study Areas	
	Mongla	Shyamnagar
**Age group (%)**	***n*=143**	***n*=117**
14–17	0	1.7
18–35	41.3	38.5
35–60	51.0	56.4
60+	7.7	3.4
**Educational Qualification (%)**	***n*=143**	***n*=117**
No formal education	30.8	47
Primary	40.6	37.6
Secondary	23.1	14.5
Higher Secondary	2.8	0.9
Graduation/Post-graduation	2.8	0
**Mean Monthly Household Income (%)**	***n*=143**	***n*=117**
Below BDT 5000	19.6	23.1
5000 - 10,000 BDT	36.4	31.6
10,001 - 20,000 BDT	32.9	35
20,001 - 30,000 BDT	10.5	7.7
30,000+	0.7	1.7
No Idea	0.0	0.9
**Occupation (%)**⁎	***n*=143**	***n*=117**
Cultivation in agricultural land	0.7	0
Agricultural Labourer	0.7	6.0
Non-agricultural Labourer	6.3	24.8
Fishing	58.7	41.9
Housewife	54.5	57.3
Other	13.3	12.0

⁎ Multiple response questions.

### Recent changes and occurrence of climate change-induced natural hazards

Most of the questionnaire survey respondents in both study areas (92.3 % in Mongla and 94.0 % in Shyamnagar) opined that the occurrence rate of specific climate change-induced natural hazards has increased over the years at their locality (Table 2). According to the respondents, climate change-induced natural hazards that affected their lives and livelihoods the most are cyclones (86.0 %), salinity (66.4), and riverbank erosion (62.9 %) in Mongla. In contrast, in Shyamnagar, the percentages are cyclone (94.0 %), salinity (73.5 %), and storm surge (54.7 %). Climate change-induced natural hazards that the respondents experienced and were affected by include cyclones (88.8 %), salinity (45.5 %), riverbank erosion (44.8 %) etc. in Mongla, and cyclone (96.6 %), salinity (53.0 %), storm surge (53.0 %), etc. in Shyamnagar.

**Table 2:** Perception of recent changes and occurrences of climate change-induced natural hazards.

Variable	Study Areas	
	Mongla	Shyamnagar
**Whether the recent occurrence rate of climate change-induced natural hazards increased (%)**	***n*=143**	***n*=117**
No	7.7	4.3
Yes	92.3	94.0
No Idea	0.0	1.7
**Type of climate change-induced natural hazards that increased (%)**⁎	***n*=143**	***n*=117**
Flood/ Flash flood	35.0	34.2
Riverbank erosion	62.9	42.7
Drought	2.1	0.9
Cyclone	86.0	94.0
Storm surge	37.8	54.7
Water logging	13.3	15.4
Salinity	66.4	73.5
Tidal water intrusion/ Tidal flooding	43.4	25.6
Erratic Rainfall Pattern (Untimely rain/ low rainfall)	10.5	24.8
Extreme heat events	0.7	0.0
**Climate change-induced natural hazards that recently affected respondents (%)**⁎	***n*=143**	***n*=117**
Flood	21.7	18.8
Riverbank erosion	44.8	13.7
Drought	7.7	0.0
Cyclone	88.8	96.6
Storm surge	28.7	53.0
Water logging	15.4	19.7
Salinity	45.5	53.0
Tidal water intrusion/ Tidal flooding	26.6	23.1
Erratic rainfall pattern (Untimely rain/ low rainfall)	1.4	8.5

⁎ Multiple response questions.

### Impacts of climate change-induced natural hazards on child marriage and school dropout and women's empowerment via decision-making

Women's empowerment in household decision-making between the two locations was significantly different (Chi-squared = 6.310, *df* = 1, *P* < 0.01). Among respondents who could not participate in household decision-making, around half of the respondents in Mongla said that the occurrence of natural climate change-induced natural disasters often inhibits their contribution towards family decisions; however, the scenario is quite different in Shyamnagar where most of the respondents think natural climate change-induced natural disasters is not the cause of their inability to participate in family decision making. The occurrence of child marriage in the families under the study varied significantly between Mongla and Shyamnagar (χ2 = 17.915, *df* = 1, *P* > 0.0001) (Table 3). In Mongla, only 15.4 % of respondents have said that girls below 18 years in their family got married, while in Shyamnagar, the figure stands at 38.5 %. Only 2.1 % of respondents in Mongla confessed to giving dowry to marry off their girls. However, the percentage is relatively high in Shyamnagar, i.e., 28.2 %. About 56.6 % of respondents in Mongla and 72.8 % in Shyamnagar reported dropping out of at least one child from school in their family. In Shyamnagar, responses confirm a higher rate of dropout among girls (28.6 %) than boys (21.4 %). In many families (38.3 % in Mongla and 50 % in Shyamnagar), male and female children dropped out before completing the Secondary School Certificate (SSC) level. “*If I could complete my education, I could earn money and contribute to my family. I would then have a voice here. What I dreamt to be and where I am now!*” exclaimed an interviewee of Gabura Union of Shyamnagar, a dropout and child marriage victim. She was forcefully married off as her parents could no longer bear the cost of her education.

**Table 3:** Impact of climate change-induced natural hazards on women's decision-making, child marriage and school dropout.

Variable	Study Area	
	Mongla	Shyamnagar
**Whether women (respondents) can take part in household decision-making (%) (*χ2* = 6.310, *df* = 1, *P*>0.01)**	***n*=131**	***n*=109**
Yes	87.0	74.31
No	13.0	25.69
**If climate change-induced natural hazards inhibit women's participation in household decision-making (%)**	***n*=17**	***n*=28**
Yes	47.1	39.29
No	52.9	60.71
**Incidence of climate change-induced hazards driven child marriage in respondent's family (*χ2* = 17.915, *df* = 1, *P*>0.0001)**	***n*=143**	***n*=117**
Yes	15.4	38.5
No	84.6	61.5
**Incidence of climate change-induced natural hazards driven dowry in respondent's family (*χ2* = 36.768, *df* = 1, *P*>0.0001)**	***n*=143**	***n*=117**
Yes	2.1	28.2
No	97.9	71.8
**Incidence of climate change-induced natural hazards driven school dropout (%)**	***n*=143**	***n*=117**
Yes	56.6	71.79
No	43.4	28.21
**Dropout: Male/ Female child (%)**	***n*=81**	***n*=84**
Male child	42.0	21.4
Female child	19.8	28.6
Both	38.3	50.0

### Sufferings of the pregnant women during climate change-induced natural hazards

Around half (41 % in Mongla and 52 % in Shyamnagar) of the respondents in both study areas have reported that they or someone in their family faced some miseries during climate change-induced natural hazards due to pregnancy (Table 4). These issues include problems with movement, trouble reaching a safe place, being stuck in the climate change-induced natural hazards-affected area, unavailability of medical services and medicine, sanitation problems, and accidents while rushing to cyclone shelters. Cases have been found where pregnant women faced grave consequences as they became stranded at places where they could not avail of any emergency medical services. One such victim from Dumuria village of Gabura union, Shyamnagar, lost her twin unborn babies during the landfall of cyclone Aila. She and her family were stuck at the embankment from where they could not find any transport to take her to the hospital. After a while, her parental family rescued her and took her to the hospital, but it was too late.

**Table 4:** Difficulties caused by natural hazards for pregnant women.

Variables	Study Areas	
	Mongla	Shyamnagar
**Whether pregnant women faced problems during climate change-induced natural hazards (*χ2* = 3.975, *df* = 1, *P*>0.0**4**)**	***n*=143**	***n*=117**
Yes	40.6	52.1
No	30.8	21.4
Not applicable	28.7	26.5
**Types of problems faced by pregnant women during climate change-induced natural hazards (%)**⁎	***n*=58**	***n*=61**
Problem in movement	53.4	82.0
Trouble in reaching a safe place	84.5	90.2
Being stuck at climate change-induced natural hazards affected area	24.1	32.8
Unavailability of medical services and medicine	25.9	26.2
Sanitation problem	19.0	39.3
Faced an accident while going to the cyclone shelter	5.2	0.0

⁎ Multiple response questions.

### Displacement and related issues

45 % of respondents in Mongla and 29 % in Shyamnagar have said that their families have been displaced due to climate change-induced natural hazards at least once in their lifetime (Table 5). Climate change-induced natural hazards that caused the most displacements are riverbank erosion and cyclone in Mongla and cyclone and flood in Shyamnagar. Displacement due to climate change-induced natural hazards among the two locations was found to be significantly different (Chi-squared = 7.336, *df* = 1, *P* > 0.001), which might result from the high riverbank erosion rate in Mongla Upazila. After displacement, the most common temporary residence of people of both areas is on/along embankments or in the open air by the side of roads. After displacement, women faced some distinct difficulties while staying at their temporary residence, including sanitation problems, increased workload, unpleasant behavior by neighbors, risk of violence or sexual harassment, and being more vulnerable to climate change-induced natural hazards. Around 79 % of Mongla and around 68 % of respondents of Shyamnagar are afraid of being displaced from their current homes due to future climate change-induced natural hazards. *“We need a safe place to stay protected from the river's gulp, the devastation of cyclones, and where there will be enough water to drink. That is all we want,”* said one respondent from Kanainagar village, Chandpai Union, Mongla, Bagerhat. Monira's family is a victim of riverbank erosion. The river had already taken all their cultivable land and a portion of their house. They have been desperately trying to move further inward of the village but have not yet got any place to move to.

**Table 5:** Displacement and women's sufferings.

Variables	Study Areas	
	Mongla	Shyamnagar
**Whether the respondent's family was displaced due to climate change-induced natural hazards ever (*χ2* = 7.336, *df* = 1, *P*>0.001)**	***n*=143**	***n*=117**
Yes	45.5	29.1
No	54.5	70.9
**Reason of displacement (%)**⁎	***n*=65**	***n*=34**
Riverbank erosion	87.7	17.6
Cyclone	20.0	79.4
Storm surge	1.5	0.0
Flood	4.6	58.8
Salinity	1.5	2.9
**Issues faced by women after displacement (%)**⁎	***n*=44**	***n*=28**
Victims of violence or eve-teasing	2.3	11.8
Risk of violence or eve-teasing	6.8	2.9
Bad behavior by neighbors	25.0	41.2
Sanitation Problem	79.5	73.5
Risk of human trafficking	0.0	0.0
Increased workload	45.5	38.2
Lack of drinking water	2.3	2.9
Lack of food and clothing	2.3	0.0
**Possibility of future displacement due to climate change-induced natural hazards (*χ2* = 4.519, *df* = 2, *P*>0.1)**	***n*=143**	***n*=117**
Yes	79.0	67.5
No	6.3	8.5
No Idea	14.7	23.9

⁎ Multiple response questions.

### Health issues of women

Women in the study areas have been suffering from some dire health conditions linked with the water salinity as they perceive it. In both Upazilas, women who usually drink salt water or water of poor quality (from ponds or rivers) often suffer from diarrhea (83.1 % in Mongla and 86.32 % in Shyamnagar) and hypertension (25.4 % in Mongla, 46.15 % in Shyamnagar) (Table 6). Due to constant use of and contact with saline water, women are found to suffer severe skin diseases (69.7 % in Mongla and 92.31 % in Shyamnagar), hair fall (33.8 % in Mongla, 41.88 % in Shyamnagar), and skin burn/ darkening (reported by 42.3 % women in Mongla and 35.04 % women in Shyamnagar). In both study areas, two age groups are found to be suffering most from the health issues, namely, children aged below 14 years (36.36 % in Mongla and 34.20 % in Shyamnagar) and 30–50-year-old adults (34.97 % in Mongla and 37.60 % in Shyamnagar). Again, the results show that female members of the respondents’ families suffer more from different ailments than their male counterparts (66.4 % in Mongla and 71.8 % in Shyamnagar). Most respondents (61.5 % in Mongla and 68.4 % in Shyamnagar) said that the female members of the family suffered from some distinct feminine health issues. As many respondents were fisherwomen who wade into waist-deep water and catch fingerlings in the rivers, they have reported several reproductive health problems that they perceive are attributable to the use and involvement with saline water of rivers or ponds. 64.8 % of the responding women in Mongla and 53.8 % in Shyamnagar have been suffering from infection or inflammation in reproductive organs (i.e., vagina, uterus, fallopian tubes, and ovaries). Some of the other prominent health issues found among the respondents are difficulties with menstrual hygiene management (36.4 % in Mongla and 40.0 % in Shyamnagar), hypertension during pregnancy (19.3 % in Mongla and 22.5 % in Shyamnagar), pre-eclampsia/ eclampsia (23.9 % in Mongla and 11.3 % in Shyamnagar), miscarriage (11.4 % in Mongla and 20.0 % in Shyamnagar) and urinary tract infection (10.2 % in Mongla and 1.3 % in Shyamnagar) etc. Several women (1.1 % in Mongla and 12.5 % in Shyamnagar) have lost their uterus (removed by surgery) due to prolonged bleeding, infection, or tumor. A fisherwoman (57) from Kanainagar Village, Chandpai, Mongla, Bagerhat, stated that she had experienced miscarriage four times due to her constant contact with saline water (as she perceives). She also has itching and an infection in her uterus. She even collected fingerlings wearing sarees during her pregnancy as she had no other livelihood option in her area then. During her fourth pregnancy, her water-breaking (leakage of amniotic fluid) started when she was gleaning fingerlings. She was admitted to the local hospital for four days and was bedridden for over a month after being brought back home. Various economic and non-economic losses and damages due to frequent climate change-induced natural hazards are causing mental stress among the inhabitants of the study areas. Concerning the gender group suffering most from such mental strain, most of the respondents from Mongla (50.3 %) said that women exclusively suffer the most. In contrast, the observation of respondents from Shyamnagar is different. Most of Them (50.4 %) think both male and female household members suffer equally from mental stress.

**Table 6:** Health problems of women.

Variables	Study Areas	
	Mongla	Shyamnagar
**Health issues attributable to climate change-induced natural hazards (%)**⁎	***n*=142**	***n*=117**
Diarrhea	83.1	86.32
Skin burns/ Darkening	42.3	35.04
Skin diseases	69.7	92.31
Hair fall	33.8	41.88
Hypertension	25.4	46.15
Loss of organ	0.0	0.85
Physical injury	28.9	34.19
**Age group suffering most from health issues (*χ2* = 26.406, *df* = 4, *P*>0.0001)**	***n*=142**	***n*=117**
<14	36.36	34.20
14–18	2.10	20.50
30–50	34.97	37.60
50+	16.08	7.70
No specific age group	10.49	0.00
**Gender group suffering most from health issues (*χ2* = 0.876, *df* = 2, *P*>0.6)**	***n*=142**	***n*=117**
Female Member	66.4	71.8
Male Member	14.7	12.0
Both	18.9	16.2
**Whether women members of the respondent's family faced any specific health issues that men didn't face (*χ2* = 1.316, *df* = 1, *P*>0.2)**	***n*=143**	***n*=117**
Yes	61.5	68.4
No	38.5	31.6
**Women-specific health issues (%)**⁎	***n*=88**	***n*=80**
Infection/ Inflammation in reproductive organs (vagina, uterus, fallopian tubes, ovaries)	64.8	53.8
Problems in menstrual hygiene management	36.4	40.0
Hypertension during pregnancy	19.3	22.5
Pre-eclampsia/ Eclampsia	23.9	11.3
Miscarriage	11.4	20.0
Urinary Tract Infection	10.2	1.3
Abdominal pain	1.1	0.0
Anemia	22.2	0.0
Breast tumor	1.1	0.0
Irregular menstruation	1.1	2.5
Low pressure	1.1	0.0
Tumor like growth in breast	1.1	0.0
Uterus tumor	3.4	0.0
Uterus tumor operation (uterus removed)	1.1	12.5
**Gender group having the most** m**ental sufferings (*χ2* = 4.4026, *df* = 2, *P*>0.1)**	***n*=137**	***n*=114**
Female members	51.8	39.5
Male members	8.0	7.9
Equally both	40.1	52.6

⁎ Multiple response questions.

### Drinking water sources and related issues

Mongla and Shyamnagar are affected by salinity, and the inhabitants suffer from scarcity of fresh water. Significant effort must be made to procure drinking water for the family. According to the respondents, they use various sources for their daily drinking water collection. Mostly noted sources are- household rainwater harvesting systems (72.7 % in Mongla; 68.4 % in Shyamnagar), Unpaid freshwater ponds which collect and store rainwater and often equipped with a Pond Sand Filtration system (54.5 % in Mongla; 54.7 % in Shyamnagar), Paid Pond water/ Pond water bought from vendors (38.5 % in Mongla; 34.2 % in Shyamnagar), River water (42.7 % in Mongla; 6.8 % in Shyamnagar), Governmental/ non-governmental water supply system (9.8 % in Mongla; 19.7 % in Shyamnagar) etc. (Table 7). Some people in Mongla (12.6 %) use a unique source for drinking water: a makeshift dug well made into sandy land where fresh water is accumulated through seepage from underground.

**Table 7:** Drinking water sources and women's burden.

Variables	Study Area	
	Mongla	Shyamnagar
**Source of drinking water (%)**⁎	***n*=143**	***n*=117**
Rainwater harvesting (own/ unpaid)	72.7	68.4
Rainwater harvesting (paid)	5.6	0.0
Water from pond (paid/ bought from vendors)	38.5	34.2
Water from pond (unpaid)	54.5	54.7
River	42.7	6.8
Govt./ non-governmental water supply system (Harvested rainwater in tank/ supply in the pipeline)	9.8	19.7
Water from missionary at a subsidized price	0.7	0.0
Water from the tube well	2.1	9.4
Water from the dug well	12.6	0.0
**Gender group responsible for drinking water collection (*χ2* = 9.770, *df* = 2, *P*>0.001)**	***n*=132**	***n*=96**
Female member	62.9	78.1
Male member	6.8	8.3
Both	30.3	12.5
Missing data	0.8	1.0
**Whether women faced any issues while collecting water (*χ2* = 0.279, *df* = 1, *P*>0.5)**	***n*=143**	***n*=117**
Yes	44.1	49.6
No	42.0	41.0
Not Applicable	14.0	9.4
**Issues faced by women while collecting water**	***n*=63**	***n*=58**
Angry/abusive behavior by peers/ owner of the source	57.14	75.86
Sexual harassment on the way or at the source	9.52	10.34
Physical illness/ injury while transporting the water	69.84	67.24
Fear/incident of an accident on the way	3.17	0.00

⁎ Multiple response questions.

This study has found that most women (62.9 % in Mongla and 78.1 % in Shyamnagar) are responsible for collecting water for household consumption. The responsible person for water collection in the two study areas drew a significant difference (Chi-squared = 9.770, *df* = 2, *P* > 0.001), which may be on account of male member availability at home in Mongla Upazila is higher than Shyamnagar Upazila, that's why they can take part in water collection. Around half of the respondents reported that women face various problems while collecting water, like angry/ abusive behavior from neighbors or owner of the source (57.14 % in Mongla, 75.86 % in Shyamnagar), sexual harassment on the way or at the start (almost 10 % in both areas) and physical injury or illness like body ache due to carrying heavy water pitchers for a prolonged time (just below 70 % in either location), etc. In an FGD conducted at Munshiganj, Shyamnagar, a woman said, *“Verbal altercations are very common, and even fights between peer collectors happen while fetching water. We must take water in a long queue. Teenage girls face problems like eve teasing and bad words on the way.”* Rainwater harvesting is the cheapest and the most easily accessible option for remote and isolated areas like Gabura, according to the respondents. However, not everybody can afford to buy large reservoir tanks to store enough rainwater for year-round use, and some microcredit local NGOs started a venture of selling tanks on monthly installments, that have added to the economic burden on the shoulders of poor people.

### Women's access to social safety nets

The people in this study are already underprivileged and burdened with health problems and recurrent loss and damage. Their sufferings have intensified over the years as they do not have proper access to social safety net schemes. Some respondents have reported that they are beneficiaries of some of the government social safety net programs (28.0 % in Mongla and 14.5 % in Shyamnagar) (Table 8). However, some had to pay a share of the allowance or bribe the local influencers/representatives to get registered with the government allowance. A woman in an FGD conducted in Gabura of Shyamnagar Upazila said, *“I have been trying to get a widow allowance card for the last four years. I applied for an allowance, but they said I am not old enough and would cost me BDT 5000. I can survive with that amount of money for 15–30 days. So, I stopped trying for a card. No one ever gets a card free of cost. Ward members and the Chairman both demand bribes to provide allowance cards.”*

**Table 8:** Women's access to social safety net programs.

Variables	Study Area	
	Mongla	Shyamnagar
**Whether women family members of the respondent have access to govt. social safety net scheme (*χ2* =10.806, *df* = 1, *P*>0.001)**	***n*=143**	***n*=117**
Yes	28.0	14.5
No	72.0	85.5
**Whether had to pay illegal gratification to get registered for the social safety net (*χ2* =3.292, *df* = 1, *P*>0.06)**	***n*=40**	***n*=17**
Yes	27.5	35.3
No	72.5	64.7

### Analysis of women's rights violation

Women suffer numerous problems, and their lives and livelihoods are in peril due to the ongoing crisis created by climate change-induced hazards. This study has unveiled many rights violation issues of women, which are directly or indirectly propelled by both sudden and slow onset events. Table 9 analyses how women's rights are violated, as found in the current study.

**Table 9:** Analysis of human rights violations of women in the coastal belt of Bangladesh.

Human Rights Issues	International Human Rights Instruments and Bangladesh's Law (Government of the People's Republic of Bangladesh, 1972; UNHRC, 1948; United Nations, International Covenant on Economic, Social, and Cultural Rights, OHCHR, 1966; United Nations, 1966; United Nations, 1989; United Nations, 1979)	Reasons for Rights Violations
Right to life, liberty, and security	Article 3, UDHR; Article 6, ICCPR; Article 32, Constitution of Bangladesh	• Death and injuries caused by sudden onset events like cyclones, storm surges, and floods violate women's right to life. • Climate change-induced climate change-induced natural hazards cause displacement, and displaced people become prone to violence and harassment, violating their right to liberty and social security, among others.
Right to self-determination	Article 1, ICCPR, ICESCR; Article 19,39, the Constitution of Bangladesh	• Climate change-induced natural hazards (e.g., river bank erosion, cyclones, storm surges, etc.) make women more vulnerable regarding economic, social, and cultural aspects of their lives. Climate change-induced natural disaster events exaggerate existing inequality between men and women. • Riverbank erosion and cyclones cause women to become destitute, and they cannot raise their voices due to their economic condition and social position. • Displaced women rarely get political rights in a newly settled location • Climate change-induced natural hazards cause poverty, unemployment, and sickness that prevent women from participating in decision-making in the family and the broader social context.
Right to means of subsistence	Article 25, UDHR; Article 11, ICESCR; Article 11, CEDAW; Article 15,18, 40, the Constitution of Bangladesh	• Damage and destruction of houses is a regular occurrence for people living in the studied areas. • After being displaced due to losing homestead to climate change-induced natural hazards, many people are living in makeshift houses built on *Khas* land by embankments or roads where they cannot meet necessities of life, i.e., food, clothing, shelter, education, and medical care. • Hunger, disease susceptibility, loss of income, and displacement are tangible effects of frequent and severe climate change-induced natural hazards brought on by climate change. • In salinity-affected coastal areas, women are bound to choose professions such as fishing in coastal rivers, working in saltwater shrimp farms, etc., which make them vulnerable to various health hazards, in the worst cases, threaten their lives (being attacked by crocodiles or the Royal Bengal tiger). • Due to economic constraints and social obligations, they can not change their profession at their will. • Climate change-induced natural hazards often cause them to lose work and face economic hardship due to unemployment.
Right to health	Article 12, ICESCR; Article 15(a), Article 18(a) of the Constitution of Bangladesh	• In salinity-affected southwest coastal areas, many women suffer from deadly health issues, especially infection/ disease of their reproductive organs, which often impair their reproductive health. Women who are victims of such illness associate it with prolonged exposure to saline water (through fishing in the river, using salty water in bathing, and other hygiene-related activities, etc.
(Women's) Right to water and sanitation	Article 14(2(h)), CEDAW; Article 15a of Bangladesh's constitution	• Women in study areas were found to be deprived of fresh water for cooking, drinking, bathing, and maintaining hygiene due to excessive salinity in water • Still, the sanitation facilities are rudimentary, and women struggle to maintain proper menstruation hygiene
Right to education	Article 26, UDHR; Article 13, ICESCR; Article 10, CEDAW; Article 28, CRC; Article 17 of Bangladesh's constitution	• Climate change-induced natural hazards cause permanent and temporary school dropouts of male and female children in study areas.
Right to Social Security	Article 22, UDHR; Article 15 of Bangladesh's constitution	• Women often face harassment or violation after resettling in a new location after displacement due to a cyclone or riverbank erosion. Besides, harassment while collecting water is also common, violating the right to social security.
Right to property	Article 17, UDHR; Article 42, the Constitution of Bangladesh	• Loss and damage of property are widespread in the study areas. Cyclone, storm surge, and riverbank erosion often destroy or engulfs assets of people, including houses, cultivable and homestead land, standing crops, fishing boat, net, etc.
Right to marriage with consent	Article 16, UDHR; Art. 16, CEDAW	• Climate change-induced natural hazards in these areas cause poverty, and parents consider child marriage an option for transferring risk, considering female kids a burden. Girls can rarely freely choose a spouse or enter marriage with full consent.
Rights of Rural Women	Article 14(1 and 2 (a-h)), CEDAW	• Both study areas represent rural communities of coastal Bangladesh. Women in these areas reported not having enough healthcare facilities, especially during climate change-induced natural hazards they suffer the most. Family planning and birth control services are not adequate. • Local transport and communication systems are not women-friendly. Most roads are muddy or brick-made, significantly damaged by frequent climate change-induced natural hazards. Yet women must travel through these roads daily to collect drinking water.

## Discussion

The study's findings demonstrate the significant impact of climate change-induced natural hazards on the lives and livelihoods of women living in Mongla and Shyamnagar areas, considering the increasing frequency and intensity of climate change-induced natural hazards as reported in IPCC (Intergovernmental Panel on Climate Change (IPCC), 2022), (Vineis et al., 2011) and (Haines et al., 2006). The increase in the occurrence rate of climate change-induced natural hazards reported by the respondents highlights the need for effective climate change-induced natural disaster management and preparedness measures. The study also reveals the differences in women's empowerment between the two areas, which can be attributed to the variation in their engagement in income-generating activities, which also coincided with the conclusions of the investigation carried out by (Al Mamun and Hoque, 2022), (Ghosh et al., 2021) and (Kabir and Marković, 2019).

Access to clean water is a fundamental necessity for human life. The study highlights the various difficulties women face in accessing safe drinking water, like angry/abusive behavior from neighbors or the source owner. The microcredit local NGOs’ venture of selling climate change adaptation strategies (Johnson et al., 2019; Di Falco and Sharma, 2018; United Nations Development Programme (UNDP), 2018; Fenton et al., 2017; Caretta, 2014), like rainwater harvesting tanks on monthly installments, may add to the economic burden on poor people, and there is a need for more sustainable solutions to address this issue recommended by (Jordan, 2021).

The result indicates that pregnant women and their families face significant difficulties during climate change-induced natural hazards in the study areas. The problems include mobility, lack of access to medical facilities and medicines, sanitation problems, and accidents while rushing to cyclone shelters. The situation is particularly alarming, leading to severe consequences like miscarriage. It highlights the need to improve the climate change-induced natural disaster preparedness and response system in the study areas to ensure pregnant women's and their families’ safety.

Nearly half of the respondents in Mongla and about one-third in Shyamnagar have experienced displacement due to climate change-induced natural hazards, mainly riverbank erosion, cyclones, and floods. Women faced challenges like sanitation problems, increased workload, and the risk of violence or sexual harassment while staying at temporary residences. Most respondents in both areas are afraid of future displacement, and they seek a safe place protected from climate change-induced natural hazards and access to safe drinking water. However, research output from Islam and Hasan (2016) states both pull and push factors are responsible for displacement. The results of this study may be compared to two earlier ones, such as (Mallick and Vogt, 2014) and (Tacoli, 2009).

According to this research, women in the study areas suffer from various health conditions linked to the salinity of the water they consume. In both Mongla and Shyamnagar, women who drink salt water or water of poor quality from ponds or rivers often suffer from diarrhea and hypertension. While only a few studies directly link water salinity to diarrhea, people in Bangladesh have reported experiencing these health issues, dysentery, and indigestion due to consuming water with high salt content (Jabed et al., 2018; Rahman et al., 2017). On the other hand, most studies suggest that high salinity levels in water are associated with adverse health impacts such as high blood pressure. Still, a few studies have reported conflicting results. One study in Chicago found that high salinity exposure did not significantly affect systolic blood pressure (Hallenbeck et al., 1981), while another study in Arizona found no association between salinity levels and blood pressure (Welty et al., 1986).

Additionally, constant use of and contact with saline water can cause severe skin diseases, hair fall, and skin burn/darkening; similar issues have also been reported by (Jabed et al., 2018), (Rahman et al., 2017), (Talukder et al., 2016), (Khan et al., 2011). The percentage of women affected by these conditions was higher in Shyamnagar than in Mongla. Exposure to saline water has a detrimental effect on the reproductive health of fisherwomen, as indicated by the significant prevalence of reproductive health problems. More than half of the population is affected by inflammation or infection in reproductive organs like the uterus, fallopian tubes, ovaries, and vagina, which is alarming news. Effective measures are necessary to address these issues, such as providing clean water sources and healthcare facilities.

Moreover, there are challenges associated with accessing government social safety net programs, with respondents reporting having to pay bribes or share their allowance. These issues highlight the importance of transparent and accessible government programs that can effectively address the needs of vulnerable populations.

(Naser et al., 2019), (Atkinson and Bruce, 2015), and (Tschakert and Machado, 2012) have all stated that climate change affects not only the economic and health conditions of people but also contributes to various violations of human rights. This study discusses ten specific human rights violations that women experience because of climate change in the coastal area of Bangladesh.

## Conclusion and policy recommendations

The study found that the most devastating natural hazards affecting the lives and livelihoods of women in these areas were cyclones, salinity, riverbank erosion, and storm surges. Due to their reduced access to economic opportunities, healthcare, and education due to these hazards, women in these places are especially vulnerable to the effects of natural hazards. Their ability to understand and respond to problems connected to climate change-induced natural hazards, like child marriage and dowry, is constrained. Between the two study locations, there were considerable differences in the involvement of women in household decision-making, which might be due to the difference in the rate of engagement of surveyed women in income-generating activities. With the right skills and information, people can pursue income-generating activities, support their families and communities, and improve their ability to cope with climate change-induced natural hazards. However, women face challenges during climate change-induced natural hazards related to pregnancy and childbirth, underscoring the importance of providing adequate healthcare services to pregnant women during climate change-induced natural hazards.

In the same way, women suffer from reproductive health problems, especially those who are related to fishing. The effects of climate change on health, particularly reproductive health in coastal areas of Bangladesh, could be studied further using additional medical diagnostic procedures. Without a doubt, women's rights are violated in these two study areas, which needs greater attention from the Bangladesh government and other international women's rights protection organizations. This study recommends the following issues for improving women's condition in the coastal belt.

· To keep women away from saline water, new ventures of innovative livelihood options should be introduced that can be gained through proper craftsmanship training or by providing enough support to grow small and medium enterprises.
· To ensure access to the social safety net of coastal women, the process should be made more public-oriented. For instance, community-based committees can be formed to choose suitable people for the safety net programs.
· Cyclone shelters in the coastal belt must be upgraded with enough water and sanitation facilities, especially for women.
· Women should be supported with enough infrastructure to store rainwater. It can be done on a cluster basis.

## Limitation

The study was carried out with a small number of participants. Moreover, the focus is only on some vulnerabilities and women's rights violations. Hence, many issues couldn't be explored, which can be considered future research scope. Lastly, the study did not involve many teenagers (who were also exposed to different problems), which can be another limitation.

## Author contribution statement

All authors contributed to the study's conception and design, although the basic idea for this research sprang from the minds of Md Shamsuddoha and Md. Akib Jabed. Md Shahnul Islam, Naznin Sultana, Al Imran, Sheikh Nur Ataya Rabbi, Tanje Un Jenat, Shanjia Shams, and Mehoraf Sharif performed material preparation, data collection, and analysis. The first draft of the manuscript was written by Md. Akib Jabed, Md Shahnul Islam, and Naznin Sultana, and all authors commented on the draft versions. Md Shamsuddoha did the final revision. All authors read and approved the final manuscript.

## Ethical approval

This study was ethically reviewed and approved by the CPRD Ethical Review Committee (Memo No. CPRD/CJ/2023–001).

## Consent to participate

Regarding the consent, we made sure every one of our participants gave informed consent. We prepared a statement of support, which each interviewer carried with them. As many participants could not read with full comprehension, we verbally communicated the message. A local guide spoke to the participants in the local dialect to ensure they fully comprehended the statement and voluntarily gave consent. For participants below 18, approval was taken from their legal guardians (parents).

## Declaration of competing interest

The authors declare the following financial interests/personal relationships which may be considered as potential competing interests:

Md Shamsuddoha, Md. Akib Jabed, Md Shahnul Islam, Naznin Sultana, Al Imran, Sheikh Nur Ataya Rabbi, Tanje Un Jenat, Shanjia Shams, and Mir Mehoraf Sharif report that financial support, administrative support, and travel were provided by Bread for the World and Diakonia.

## Appendix Supplementary materials

Image, application 1

Image, image 2
