# Supplementary material for: Impacts of climate change-induced natural hazards on women and their human rights implications: A study in the southwest coast of Bangladesh
Source: J Migr Health. 2024 Mar 6;9:100221. doi: 10.1016/j.jmh.2024.100221 (PMC10946321; doi:10.1016/j.jmh.2024.100221)
Supplement: Supplementary file 1 [file mmc1.docx]

**Informed Consent Form (English)**

**Research Title:** Climate Change Impacts on Women and Their Implications for Human Rights Violations: A Case Study in the South-West Coast of Bangladesh

**Researcher/ Interviewer:** ________________________________

**Organization:** Center for Participatory Research and Development

**Date of Consent:** _____________________

I, ________________________, hereby consent to participate in the research project titled “Climate Change Impacts on Women and Their Implications for Human Rights Violations: A Case Study in the South-West Coast of Bangladesh” conducted by _________________________ from Center for Participatory Research and Development. I have been provided with information about the research and my role as a participant. I understand the purpose, procedures, potential risks, and benefits associated with my involvement in this study.

**Project Description:** The project titled as “Research and Advocacy to enhance climate justice for LDCs, Bangladesh in Particular under the Paris Climate Agreement”, aims to ensure due consideration of gender-specific and indigenous peoples’ rights in national and global discourses of loss and damages and forced migration/displacement. To this end, this project will generate/provide knowledge to promote advocacy activities focused on evidence-based arguments, establish a knowledge-based CSO platform to ensure the human rights of women and indigenous communities, and engage more young women in policy-advocacy activities. The project further aims to establish adaptation failure scenarios to aid global negotiations and national responses to climate change-induced loss and damage measures.

**Participant's Role:** I understand that my role in this study involves participating in Questionnaire Survey/ FGD/ KII/ Case Study/ In-depth Interview/ Other type of data collection procedure, which may include the following:

1. **Photography:** I consent to being photographed during the research process. These photographs may be used in research presentations, reports, and publications related to the project.
2. **Audio and Video Recording:** I consent to audio and video recordings of interviews, discussions, or interactions related to the research. These recordings may be used for analysis, presentations, reports, and publications.
3. **Quoting:** I consent to my statements and contributions being quoted in research outputs, including presentations, reports, and publications. Pseudonyms or initials may be used to protect my identity.

**Confidentiality and Anonymity:** I understand that my identity will be kept confidential to the extent permitted by law and research ethics. My real name will not be used in any publications or presentations. Any personal information that could identify me will be pseudonymized or removed.

**Withdrawal of Consent:** I understand that participation is voluntary, and I have the right to withdraw from the study at any time without penalty or loss of benefits. I can withdraw my consent for the use of my photographs, recordings, or quotes even after the data collection has taken place.

**Data Use and Dissemination:** I understand that the data collected from my participation may be analysed and used for research purposes. Research outputs may include presentations, reports, and publications. However, my privacy will be protected, and my data will be used in a manner that ensures anonymity.

**Questions and Contact Information:** I have had the opportunity to ask questions about the study and my participation. I understand that I can contact [Interviewer's Name] at [Contact Information] if I have any further questions or concerns.

**Voluntary Consent:** I have read and understood the information provided in this consent form. I voluntarily agree to participate in the research project under the conditions described above.

Participant's Signature: ______________________________ (Date: ____________)

**Parent/ Guardian Consent (If Participant is a Minor):** I, as the parent/legal guardian of the participant named above, have read and understand this consent form. I hereby give my permission for my child to participate in the research project as described.

Parent/Guardian Signature: ______________________________ (Date: ____________)

**Informed Consent Form (Bangla)**

গবেষণা শিরোনামঃ নারীদের উপর জলবায়ু পরিবর্তনজনিত প্রভাব এবং মানবাধিকার লঙ্ঘন : বাংলাদেশের দক্ষিণ-পশ্চিম উপকূলে একটি সমীক্ষা

**গবেষক/ সাক্ষাতকার গ্রহণকারীঃ ______________________________________________________**

**প্রতিষ্ঠানঃ** সেন্টার ফর পার্টিসিপেটরি রিসার্চ এন্ড ডেভেলপমেন্ট-সিপিআরডি

**সম্মতি প্রদানের তারিখঃ ________________________________________________**

আমি, __________________________________, সেন্টার ফর পার্টিসিপেটরি রিসার্চ এন্ড ডেভেলপমেন্ট-সিপিআরডি -এর অধীনে __________________________________ দ্বারা পরিচালিত “নারীদের উপর জলবায়ু পরিবর্তনজনিত প্রভাব এবং মানবাধিকার লঙ্ঘন : বাংলাদেশের দক্ষিণ-পশ্চিম উপকূলে একটি সমীক্ষা” শীর্ষক গবেষণায় অংশগ্রগণের জন্য সম্মতি দিচ্ছি। আমাকে উক্ত গবেষণা এবং অংশগ্রহণকারী হিসেবে আমার ভূমিকা সংক্রান্ত তথ্য প্রদান করা হয়েছে। এই গবেষণায় যুক্ত থাকার উদ্দেশ্য, কার্যপ্রণালী, সম্ভাব্য ঝুঁকি এবং উপকারিতা সম্পর্কে আমি সম্যকভাবে অবগত আছি।

**প্রকল্প বর্ণনাঃ** এই গবেষণা প্রকল্পটির উদ্দেশ্য জলবায়ু পরিবর্তন-জনিত ক্ষয়ক্ষতি এবং বাধ্য-অভিবাসন/ স্থানান্তরণ সংক্রান্ত জাতীয় এবং বৈশ্বিক আলোচনায় লিঙ্গ-নির্দিষ্ট এবং আদিবাসী জনগোষ্ঠীর অধিকার যথাযথ বিবেচনা নিশ্চিতকরণ। এই লক্ষ্যে মানবাধিকারের আবশ্যকীয় প্রচার এবং নারী ও আদিবাসী সম্প্রদায়ের অধিকার নিশ্চিত করতে প্রমাণ-ভিত্তিক ও যুক্তি-নির্ভর অধিপরামর্শমূলক জ্ঞান উৎপাদন/ প্রদান করা, নারী ও আদিবাসী সম্প্রদায়ের মানবাধিকার নিশ্চিত করার পাশাপাশি জাতীয় ও বৈশ্বিক পলিসি স্টেকহোল্ডারদের প্রভাবিত করার জন্য একটি জ্ঞান-ভিত্তিক সিএসও (CSO) প্ল্যাটফর্ম প্রতিষ্ঠা করা, তরুণ কর্মজীবী নারী ও নারী শিক্ষার্থীদের নীতি-অধিপরামর্শমূলক কার্যক্রমে সংযুক্ত করা এবং জলবায়ু-জনিত ক্ষয়ক্ষতি মোকাবেলায় বিস্তৃত পরিসরে পদক্ষেপ (যেমন সামাজিক সুরক্ষা জাল, পরিকল্পিত পুনর্বাসন, বাস্তুসংস্থান পুনরুদ্ধার ইত্যাদি) গ্রহণের প্রতি বৈশ্বিক আলোচনা ও জাতীয় প্রতিক্রিয়ায় সহায়ক হিসেবে অভিযোজন ব্যর্থতার দৃশ্যকল্প প্রতিষ্ঠা করা এই প্রকল্পের উদ্দেশ্য।

**অংশগ্রহণকারীর ভুমিকাঃ** গবেষণায় আমার ভূমিকা প্রশ্নোত্তর জরিপ/ ফোকাস গ্রুপ আলোচনা/ মূল তথ্যদাতা সাক্ষাতকার/ বিশদ সাক্ষাতকার/ অন্যান্য যেকোন প্রকার তথ্য সংগ্রহ পদ্ধতি প্রভৃতিতে অংশগ্রহণ করা, যার মধ্যে নিম্নলিখিত বিষয়াবলি অন্তভুর্ক্ত হতে পারেঃ

**১। আলোকচিত্রঃ** আমি গবেষণা পদ্ধতির অংশ হিসেবে আমার আলোকচিত্র গ্রহণে সম্মতি জানাচ্ছি। এই ছবি গবেষণা সংক্রান্ত উপস্থাপনা, প্রতিবেদন এবং প্রকাশনায় ব্যবহৃত হতে পারে।

**২। অডিও ও ভিডিয়ো ধারণঃ** আমি গবেষণা সংক্রান্ত সাক্ষাতকার, আলোচনা এবং মিথষ্ক্রিয়ায় নিজ অডিও ও ভিডিয়ো ধারণে সম্মতি জানাচ্ছি। এই ধারণকৃত তথ্য বিশ্লেষণ, উপস্থাপনা, প্রতিবেদন এবং প্রকাশনায় ব্যবহৃত হতে পারে।

**৩। উদ্ধৃতিঃ** গবেষণা ফলাফল যেমন উপস্থাপনা, প্রতিবেদন এবং প্রকাশনায় আমার বক্তব্য এবং অবদান উদ্ধৃতকরণে সম্মতি জানাচ্ছি। আমার নিরাপত্তার স্বার্থে ছদ্মনাম অথবা আদ্যক্ষর ব্যবহৃত হতে পারে।

**গোপনীয়তা ও বেনামীতাঃ** আইন ও গবেষণা নীতি অনুসারে আমার পরিচয় গোপন রাখার ব্যাপারে আমি অবগত আছি। আমার প্রকৃত নাম কোন প্রকাশনা অথবা উপস্থাপনায় ব্যবহার হবেনা। সনাক্তকারীর/ তথ্যদাতার যেকোন ধরনের তথ্য ছদ্মনামকরণ করা অথবা মুছে ফেলা হবে।

**সম্মতি প্রত্যাহারঃ** আমার অংশগ্রহণ ঐচ্ছিক এবং কোন ধরনের শাস্তি অথবা ক্ষতিপূরণ ছাড়া গবেষণার যেকোন পর্যায়ে আমি আমার বক্তব্য প্রত্যাহারের অধিকার সম্পর্কে অবগত আছি। এমনকি তথ্য সংগ্রহ সম্পন্নের পরেও আমার ছবি, রেকর্ডিং, অথবা উদ্ধৃতি ব্যবহারের ক্ষেত্রে আমি সম্মতি প্রত্যাহার করতে পারি।

**তথ্য ব্যবহার ও বিতরণঃ** আমার অংশগ্রহণ থেকে প্রাপ্ত তথ্য গবেষণার উদ্দেশ্যে বিশ্লেষণ ও ব্যবহার সম্পর্কে আমি অবগত আছি। গবেষণা ফলাফলে উপস্থাপনা, প্রতিবেদন এবং প্রকাশনা অন্তর্ভুক্ত থাকতে পারে। এক্ষেত্রে আমি এই মর্মে আশ্বস্ত যে, আমার নিরাপত্তা সুরক্ষিত থাকবে এবং আমার তথ্য এমনভাবে ব্যবহার করা হবে যাতে তথ্যের নামহীনতা নিশ্চিত হয়।

**প্রশ্ন এবং যোগাযোগ তথ্যঃ** আমি এই গবেষণা এবং আমার অংশগ্রহণ সম্পর্কে প্রশ্ন করার সুযোগ পেয়েছি। আমি পরবর্তীতে কোন প্রশ্ন অথবা ভাবনা নিয়ে ________________________________এর সাথে যোগাযোগের ব্যাপারে অবগত আছি।

**ঐচ্ছিক সম্মতিঃ** এই সম্মতি পত্রে লিখিত তথ্যগুলো আমি পড়েছি এবং যথাযথভাবে অবগত আছি। উপর্যুক্ত শর্তাবলীর অধীনে আমি ঐচ্ছিকভাবে এই গবেষণায় অংশগ্রহণ করেছি।

অংশগ্রহণকারীর স্বাক্ষরঃ _____________________________________________ (তারিখঃ_____________________________________ )

**পিতামাতা/অভিভাবক এর সম্মতি (যদি অংশগ্রহণকারী নাবালক/নাবালিকা হন)ঃ** আমি, উপরে উল্লেখিত অংশগ্রহণকারীর পিতামাতা/অভিভাবক হিসেবে এই সম্মতিপত্র পড়েছি এবং এতে লিখিত তথ্য সম্পর্কে অবগত আছি।

পিতামাতা/অভিভাবক এর স্বাক্ষরঃ ______________________________________ (তারিখঃ ____________________________________)
