# Supplementary material for: Impacts of climate change-induced natural hazards on women and their human rights implications: A study in the southwest coast of Bangladesh
Source: J Migr Health. 2024 Mar 6;9:100221. doi: 10.1016/j.jmh.2024.100221 (PMC10946321; doi:10.1016/j.jmh.2024.100221)
Supplement: Supplementary file 2 [file mmc2.docx]

**Questionnaire Survey to Assess Vulnerability (Risk and Loss and Damage) of Women due to Climate Change**

Questionnaire serial no: ………………. Name of the interviewer: ……………………...........................

Date: …………………………….....

Village: ………………… Union: ………………… Upazila: ............................... District: ………………

**1. Information on personal and socioeconomic status**

1.1) Name of the respondent: … … … … … … … … … … Contact no: … … … … … … … … … …

1.2) Age: a. 🞎 14-17 b. 🞎 18-35 c. 🞎 36-60 d. 🞎 60+

1.3) Sex: a. 🞎 Male b. 🞎 Female c. 🞎 Others

1.4) Education: a. 🞎 Illiterate b. 🞎 Primary c. 🞎 Secondary d. 🞎 Higher Secondary

e. 🞎 Graduation/ Post graduation

1.5) Status of the respondent in the family:

a. 🞎 Earning Member (male/female) b. 🞎 Wife of the earning member

c. 🞎 Son/daughter of the earning member d. 🞎 Elderly people of the family

1.6) Total number of the family member …………

1.7) Number of the earning member: a. 🞎 Female: ……... b. 🞎 Male: ……..

1.8) Monthly income of the family: … … … …

1.9) Profession of the respondent (Answer may be multiple):

1) 🞎 Cultivation in the agricultural land; a. 🞎 Own land b. 🞎 Other people’s land c. 🞎 Both

2) 🞎 Agricultural labor in other’s land; Type: a. 🞎 Seasonal b. 🞎 Year-round

3) 🞎 Non-agricultural labor: … … … … … Type: a. 🞎 Seasonal b. 🞎 Year-round

4) 🞎 Fishing (own aquaculture/pond)

5) 🞎 Fisherman: a. 🞎 Fishing in other people’s pond/aquaculture

b. 🞎 Fishing in other people’s boat c. 🞎 Fishing in own boat

6) 🞎 Housewife

7) 🞎 Own business; Type of the business: ………………………………………...

8) 🞎 Job (public/private)

9) 🞎 Others: Name of the profession/type ……………………………………

1.10) Ownership of the residence a. 🞎 Government (khas land) b. 🞎 Own ownership (rented)

c. 🞎 Own ownership

1.11) Ownership of the house: a. 🞎 Rented house b. 🞎 Own house

**2. Information on climate change and disasters**

2.1) Has the degree of occurrence of climatic disasters increased in the recent times?

a. 🞎 Yes b. 🞎 No

If the answer is ***Yes***, which disaster has increased its degree of occurrence? (Answer may be multiple)

a. 🞎 Flood/Flash flood b. 🞎 Riverbank erosion c. 🞎 Drought d. 🞎 Storm e. 🞎 Cyclone

f. 🞎 Water logging g. 🞎 Salinity h. 🞎 Intrusion of the tidal flows

i. 🞎 Untimely rainfall/ light rainfall j. 🞎 Others: . . .

2.2) In which disaster you/your family have affected in recent times? (Answer may be multiple)

a. 🞎 Flood/Flash flood b. 🞎 Riverbank erosion c. 🞎 Drought d. 🞎 Storm e. 🞎 Cyclone

f. 🞎 Water logging g. 🞎 Salinity h. 🞎 Intrusion of the tidal flows

i. 🞎 Untimely rainfall/ light rainfall j. 🞎 Others: . . .

**3. Information on socioeconomic crisis**

3.1) Can you/adult female members of your family actively participate in the decision-making process in the family? (not applicable for the family that has no adult male members)

a. 🞎 Yes b. 🞎 No

If the answer in ***No***

3.2) Do **disasters** obstruct the active participation in decision-making process for you/adult female family members of your family? (not applicable for the family that has no adult male members) a. 🞎 Yes b. 🞎 No

3.2.1) If the answer is ***Yes,*** which obstructs you? (Answer may be multiple)

Due to climate change: i) Loss of profession ii) Decrease of income/financial contribution in the family

iii) Frequent illness iv) due to frequent illness iv) Engaging in disgraceful profession

v) due to increase of work pressure vi) Others: . . . . . . . . . . . . . .

3.3) Did you have married off your minor daughter to decrease expenditure due to poverty driven by disasters?

a. 🞎 Yes b. 🞎 No

3.4) Did you have to pay dowry for the marriage of any girl in your family? a. 🞎 Yes b. 🞎 No

3.4.1) If the answer is ***Yes***, mention the cause. . . . . . . . . . . . . . .

3.5) Did any of the child of your family have to stop going to school due to disasters?

| **Type of the problem** | **Number** | | **Cause** (put tick mark) | | | |
| --- | --- | --- | --- | --- | --- | --- |
|  | **Boy** | **Girl** | **Financial loss/scarcity** | **Immediate adverse situation during disaster/post-disaster period** | **Migration** | **Others** |
| **Permanently** dropout of school |  |  |  |  |  |  |
| **Temporarily** stop going to school |  |  |  |  |  |  |

3.6) What type of clothes do you and female members of your family wear in general? (Answer may be multiple)

a. 🞎 Saree b. 🞎 Salwar suit c. 🞎 Maxi d. 🞎 Others

3.7) Did you/female members of your family face any trouble (for example, trouble in moving fast) regarding clothing during disaster (flood/ storm/ cyclone)?

a. 🞎 Yes b. 🞎 No

3.7.1) If it is ***Yes***, which type of trouble have you faced? (Answer may be multiple)

a. 🞎 Swimming b. 🞎 Moving fast to a safe place during disaster c. 🞎 Carrying children

d. 🞎 Others: . .

3.8) Were you/any other female member of your family in trouble while pregnant during disaster?

a. 🞎 Yes b. 🞎 No c. 🞎 Not applicable

3.8.1) If the answer is ***Yes***, which type of trouble have you faced? (Answer may be multiple)

a. 🞎 Trouble in the movement b. 🞎 Trouble in moving to a safe place

c. 🞎 Being stuck in the disastrous place d. 🞎 Lack of access to emergency medical care and medicine e. 🞎 Sanitation problems f. 🞎 Others: … … … … … … …

3.9) Did you/your family ever have to go through forced displacement/migration due to disaster

a. 🞎 Yes b. 🞎 No

If the answer is ***Yes,***

3.9.1) In which disaster have you affected and faced forced migration/migration/displacement?

i) Riverbank erosion ii) Storm iii) Cyclone iv) Flood v) Salinity vi) Sea level rise

vii) Others: . . .

3.9.2) Where did you migrated? (Answer may be multiple)

i) Parents/relative’s house ii) Neighbor’s house

iii) Open place/ near of the embankment/ roadside area iv) Khas land of the government

v) Urban slums vi) Others: … … … … … … …

3.9.3) Where is the migrated place? (Answer may be multiple)

i) Same village ii) Same union iii) Same upazila iv) Other upazila v) Other zilla

3.9.4) Identify from the list below any problems you/your family faced while living in the changed location during forced migration/migration/displacement? (Answer may be multiple)

a. 🞎 Victim of Sexual harassment/violence b. 🞎 Risk of Sexual harassment /violence

c. 🞎 Victim of ill-treatment of the neighbor d. 🞎 Sanitation problem

e. 🞎 Risk of trafficking f. 🞎 Increase of work pressure

g. 🞎 Others: … … … … … … …

3.10) Do you think that you will be migrated in the future due to climate change impact?

a. 🞎 Yes b. 🞎 No c. 🞎 No idea

3.11) Did you/any other female member of your family have to move alone/with male member to other place in search of work?

a. 🞎 Yes b. 🞎 No

3.11.1) If the answer is ***Yes,*** what type of work have you done there?

i) Laborer in brickyard ii) Catching fingerlings iii) Rice harvester

iv) Other …………………..

3.12) Did you face any trouble when all the adult male members went to urban area/a distant place in search of work?

a. 🞎 Yes b. 🞎 No

3.12.1) If the answer is ***Yes***, what type of trouble did you face? (Answer may be multiple)

I) Pressured, threatened and attacked by locals ii) Restriction on free expression of opinion iii) Increase of work pressure iv) Insecurity/ risk of sexual harassment

v) Sexual harassment/eve teasing vi) Other…………………………………

3.13) Are you interested in going to nearby shelter house pre/ during disaster?

a. 🞎 Yes b. 🞎 No

3.13.1) If the answer is ***No*** write down the cause clearly. …………………………………………………

3.13.2) If the answer is Yes, what type of crises do you face in the shelter house during a disaster? (Answer may be multiple)

I) Scarcity of food and fresh water, inadequate sanitation facilities ii) Insecurity

iii) Non compaction of space

iv) Non-availability of emergency medical facilities and essential medicines

v) No separate room for pregnant women, women and children

vi) Forced to leave together with cattle vii) Victim of physical violence

viii) Difficulty managing menstrual cycles ix) Victim of mental violence/teasing

x) Other ……………………

3.14) How family members deal with pregnant women/disabled/elderly persons during disaster?

a. 🞎 Move to shelter/safe place b. 🞎 Keep them in the disastrous place

c. 🞎 Other: …………………

3.15) Did women/children get priority in receiving emergency kid/food/safe water/emergency services during/post disaster?

a. 🞎 Yes b. 🞎 No

**4. Information on health risk**

4.1) What type of physical illness/damage/risk are your family members most likely to experience due to climate change (increased salinity, increased temperature, frequent disasters, etc.) directly or indirectly (drinking and consumption of saline and polluted water, increased temperature, etc.)?

a. 🞎 Diarrhea b. 🞎 Burning and darkening of the skin c. 🞎 Skin diseases

d. 🞎 Hair loss e. 🞎 High blood pressure f. 🞎 Dismemberment

g. 🞎 Physical injury h. 🞎 Others . . . . . . . .

4.2) Which age group members of your family have suffered the most physical illness/damage/risk due to climate change (increased salinity, temperature rise, frequent disasters, etc.)?

a. 🞎 Children (0-14) b. 🞎 Adolescents (14-18)

c. 🞎 Middle aged women/men (30-50) d. 🞎 Elderly men/Elderly women (60+)

4.2.1) Which members of the family have suffered the most physical illness/harm/risk so far?

i) Female members ii) Male members iii) Both equally

4.2.2) What are the female members of your family due to climate change (increase in salinity, increase in temperature, frequent droughts, etc.) suffered from special physical illness/disadvantages/risks (which male members are not affected by)?

i) Uterine infection/inflammation ii) Difficulty in menstrual cycle management

iii) Gestational hypertension iv) Pre-eclampsia v) Premature abortion vi) Others: … … … … …

4.3) Are your family members suffering (/have suffered) from mental stress/depression due to frequent calamities/disaster-related financial losses?

a. 🞎 Yes b. 🞎 No

4.3.1) If the answer is ***Yes***, who is likely to suffer the most?

i) Female members ii) Male members iii) Both equally

4.4) What is the source of your drinking water? (Answer may be multiple)

a. 🞎 Rainwater harvesting b. 🞎 **Pond** i) Free of cost ii) Buying water c. 🞎 River

d. 🞎 Public/private water supply system e. 🞎 Others: . . . . . . . . . . . . . . . . . . . . . .

4.5) Do you take any purification measure (e. g., alum, boiling water) if the collected water is not drinkable?

a. 🞎 Yes b. 🞎 No

4.5.1) If the answer is ***Yes***, what measure do you take? (Answer may be multiple)

i) Use of Alum ii) Water purification tablets iii) Boiling water iv) Other: . . . . . . . . . . . . . .

4.6) Which member of the family **usually** collects water?

a. 🞎 Female members of the family b. 🞎 Male members of the family c. 🞎 Both of them

4.7) How many liters (approx) of water do you carry **at a time**? . . . . . . . . . . Litre

4.8) How far do you collect water? . . . . . . . . . feet/meters

4.9) How much time (approximate) is spent in a day to collect water? . . . . . . . . . . Minutes/hours

4.10) Has any female member of your family faced any problem in **collecting water?**  a. 🞎 Yes b. 🞎 No

4.10.1) If the answer is ***Yes***, what kind of problems faced? (Answer may be multiple)

i) Angry behavior/abuse by neighbors ii) Sexual harassment iii) Physical illness iv) Others: . . . . . . .

4.11) Have you/adolescent girls/women in your family taken birth control pills to avoid menstrual cycle during water shortage?

a. 🞎 Yes b. 🞎 No

4.11.1) If the answer is ***Yes***, what problems have they faced due to taking pills? (Answer may be multiple)

i) Loss of fertility ii) Irregular menstrual cycle iii) Becoming obese

iv) Nausea v) Dizziness/Headache vi) Loss of appetite

4.12) Has anyone in your family died in a natural disaster? a. 🞎 Yes b. 🞎 No

4.12.1) If the answer is ***Yes***, enter the number. i) Female: . . . . . . ii) Male: . . . . . . . .

**5. Information on financial risk and adaptation**

5.1) What economic losses have you/your family suffered due to climate change-induced disasters? (Answer may be multiple)

a. 🞎 Home broken/lost b. 🞎 Loss of arable land/loss of land fertility c. 🞎 Loss of cattle

d. 🞎 Loss of cash crops e. 🞎 Death of poultry f. 🞎 Reduced earnings

g. 🞎 Loss of business and income h. 🞎 Losing a job and becoming unemployed

i. 🞎 Engaging in degrading activities j. 🞎 Cessation of gainful employment due to frequent illness

k. 🞎 Being forced to change careers l. 🞎 Others: … … …

5.2) What do you do to recover financial loss? (Answer may be multiple)

a. 🞎 Loans from Mohajon (Interest.....%) b. 🞎 Dadon

c. 🞎 Loans from NGOs/ Societies (Interest.....%) d. 🞎 Borrowing from relatives/neighbours (interest free)

e. 🞎 Selling land/property f. 🞎 Spending accumulated money/wealth (ornaments)

g. 🞎 Mortgaging assets/property h. 🞎 Eating less food to reduce household expenses

i. 🞎 Engaging women in gainful/income-generating activities

j. 🞎 Engaging children in gainful/income generating activities

k. 🞎 Marriage of minor girls l. 🞎 Cultivation of Disaster Tolerant Varieties

m. 🞎 Cultivation of fast growing crops n. 🞎 Climate resilient agriculture

o. 🞎 Poultry, cattle rearing p. 🞎 Production and sale of organic fertilizers

q. 🞎 Non-agricultural labour/daily wages r. 🞎 Other: . . . . . . . . . .

5.3) Do you/any female member of your family receive (/have received) any special **allowance/incentive** from any source (Government/NGO)?

a. 🞎 Yes b. 🞎 No

5.3.1) If the answer is ***Yes***, state the source and amount. .………………………………………………

5.3.2) Whether any specific amount of these allowances is to be paid to local influentials?

i) Yes ii) No

5.3.2.1) If the answer is ***Yes***, what amount to pay? ……………………………………………………

*** * * Thank you for participating in the survey * * ***

**Survey and research conduct**: Center for Participatory Research and Development - CPRD
